# Mature B cell acute lymphoblastic leukaemia with KMT2A-MLLT3 transcripts in children: three case reports and literature reviews

**DOI:** 10.1186/s13023-021-01972-5

**Published:** 2021-07-30

**Authors:** Yinghui Cui, Min Zhou, Pinli Zou, Xin Liao, Jianwen Xiao

**Affiliations:** 1 Division of Haematology and Oncology, Children’s Hospital of Chongqing Medical University, No. 136, Zhongshan 2nd Road, Yuzhong District, Chongqing, 400014 People’s Republic of China; 2 Ministry of Education Key Laboratory of Child Development and Disorders, Chongqing, People’s Republic of China; 3 Department of Hematology, Chengdu Women’s & Children’s Central Hospital, Chengdu, People’s Republic of China; 4 National Clinical Research Center for Child Health and Disorders, Chongqing, People’s Republic of China; 5 Chongqing Key Laboratory of Paediatrics, Chongqing, People’s Republic of China

**Keywords:** Mature B cell acute lymphoblastic leukaemia, KMT2A rearrangement, Children

## Abstract

**Background:**

Mature B cell acute lymphoblastic leukaemia (BAL) is characterised by French–American–British (FAB)-L3 morphology and the presence of surface immunoglobulin (sIgM) light chain restriction. BAL is also considered as the leukaemic phase of Burkitt lymphoma (BL), in which t (8; 14) (q24; q32) or its variants are related to the myelocytomatosis oncogene (MYC) rearrangement (MYCr) is usually present. However, BAL with lysine methyltransferase 2A (KMT2A, previously called Mixed lineage leukaemia, MLL) gene rearrangement (KMT2Ar, previously called MLLr) is rare.

**Results:**

Three BAL patients with KMT2Ar were enrolled between January 2017 and November 2019, accounting for 1.37% of the B-ALL population in our hospital. We also reviewed 24 previously reported cases of BAL and KMT2Ar and analysed the features, treatment, and prognosis. Total 13 males and 14 females were enrolled in our research, and the average age at diagnosis was 19.5 ± 4.95 months old. In these 27 patients, renal, central nervous system (CNS) and skin involvement were existent in 6, 4 and 3 patients, respectively; 26 patients (26/27) showed non-ALL-L3 morphology, while one patient is ALL-L3; overexpression of CD19 was detected in most cases, negative or suspicious expression of CD20 was found in 64% of patients. KMT2Ar was reported, but MYCr was not observed. 25 patients (25/27) achieved complete remission after chemotherapy or Stem cell transplantation. The patients were sensitive to chemotherapy, prospective event-free survival (pEFS) of BAL patients with KMT2Ar who received allogeneic haematopoietic stem cell transplantation (allo-HSCT) was higher than that in patients who received chemotherapy alone (83.33% vs 41.91%).

**Conclusion:**

BAL patients with KMT2Ar had unique manifestations, including younger age at diagnosis and overexpression of CD19; expression of CD20 was rare, and MYCr was undetectable. The pEFS was higher in patients undergoing allo-HSCT than in patients undergoing chemotherapy alone.

**Supplementary Information:**

The online version contains supplementary material available at 10.1186/s13023-021-01972-5.

## Introduction

Acute lymphoblastic leukaemia (ALL) is the most common neoplasm in children, and B cell acute lymphoblastic leukaemia (B-ALL) accounts for 75–80% of all ALL cases [1]. According to the 2016 World Health Organization (WHO-2016) classification, B-ALL cases were classified into several subtypes: morphology, immunophenotype, cytogenetic, and molecular genetic characteristics [1, 2]. For instance, the immunophenotypes of B-ALL populations were classified as precursor B-ALL (pB-ALL) and mature B-ALL (BAL) by flow cytometry (FCM) [2].

The most recurrent ALL type is pB-ALL. The pB-ALL comprises 90% of B-ALL cases and is characterised by the morphologic type (French–American–British (FAB) classification systems) of ALL-L1 or ALL-L2; Flow cytometric analysis with combination of CD19, CyCD22, CyCD79a, TdT, HLA-DR and/or CD22, CD10, CD20, CyIgM, CD34 appear in pB-ALL [2–4]. Lysine methyltransferase 2A (KMT2A, previously called Mixed lineage leukaemia, MLL) gene rearrangements (KMT2Ar, previously called MLLr) are generally associated with ALL-L1/ALL-L2 pB-ALL and are present in 6% of paediatric ALL cases [2, 3]. KMT2A-AFF1 (also called MLL-AF4) and KMT2A-MLLT3 (previously called MLL-AF9) fusion gene have been reported in ALL cases, while the KMT2A-MLLT3 fusion gene is a reverse factor of ALL cases [4, 5].

BAL is uncommon for ALL patients and is characterised by FAB-L3 morphology; the presence of surface IgM (sIgM) with light-chain restriction and the absence of immature B cell antigens is typical of BAL cases [2, 6]. BAL is often associated with the translocation t(8;14)(q24;q32) or its variants; the molecular genetics of BAL is characterised by myelocytomatosis oncogene (MYC) rearrangements (MYCr), and it is considered the leukaemic phase of Burkitt lymphoma (BL) [6, 7]. MYCr is overexpressed in more than 95% of BL/BAL patients [2].

However, rare cases of BAL with KMT2Ar expression have been reported in children and adults [8, 9]. This study describes the clinical features, lab findings, treatment, and prognosis of three children with mature BAL with KMT2A-MLLT3 transcripts, and we also reviewed 24 case reports in the literature.

## Patients and methods

### Entry criteria

1. One month old to under 18 years old;
2. Bone marrow morphology diagnosed as acute mature B lymphocytic leukaemia;
3. Immunological classification is acute mature B lymphocytic leukaemia;
4. with KMT2Ar.

### Exit criteria

1. Acute lymphoblastic changes in chronic myeloid leukaemia;
2. Trisomy 21, or congenital or genetic disease accompanied by organ dysfunction;
3. Other secondary leukaemia;
4. Congenital immunodeficiency or metabolic disease;
5. Those who used glucocorticoids for 14 days or more within one month before enrollment or had any history of chemotherapy or radiotherapy within three months.

### Patients and samples collection

Three BAL patients with KMT2Ar were enrolled in our hospital study. Clinical data, including age, gender, laboratory findings, treatment, and prognosis, were obtained from the patient records and retrospectively analysed. This study was approved by the Ethics Committee of the Children’s Hospital of Chongqing Medical University (CHCMU) and Chengdu Women’s & Children’s Central Hospital (CWCCH), and written informed consent was obtained from all parents.

### Bone marrow analysis

Three bone marrow (BM) samples were obtained at diagnosis and at different time points (TP) after chemotherapy. According to morphology, immunophenotype, cytogenetic and molecular genetics, classification was performed at diagnosis according to the WHO-2016 classification of tumours of the haematopoietic and lymphoid tissues [2]. The morphologic type was classified as ALL-L1, L2 or L3 by FAB subtyping. The immunophenotype was determined by FCM with monoclonal antibody markers consisting of B cell, T cell, myeloid and stem/progenitor cell markers, and minimal residential disease (MRD) markers screened by FCM at diagnosis and monitored at different TP [1]. Chromosomal karyotyping and fluorescence in situ hybridisation (FISH) of ETV6-RUNX1, BCR-ABL, KMT2Ar, PDGFRB and MYCr and other rearrangements were performed as reported in the literature; 29 common fusion genes (dupMLL, MLL/AF4, MLL/AF6, BCR/ABL1(p190), MLL/AF1P, MLL/AFX, ALL/ENL, BCR/ABL1(p210), TCF/PBX1, TEL/AML, SIL/TAL1, TLS/ERG, E2A/HLF, TEL/ABL1, HOX11, ETV6/ABL1, NUP214/ABL1, RANBP2/ABL1, SNX2/ABL1, ZMIZ1/ABL1, RCSD1/ABL1, RCSD1/ABL2, ZC3HAV1/ABL2, PAG/ABL2, SSBP2/CSF1R, SSBP2/PDGFRβ, TNIP1TNIP1/ PDGFRβ, ZEB2/PDGFRβ, EBF1/PDGFRβ) were assayed by multiplex nested reverse transcription-polymerase chain reaction (multiplex RT-PCR) and confirmed by split RT-PCR as reported in the literature [10].

### Treatment

Patient 1 and Patient 2 were treated according to the B non-Hodgkin lymphoma 2009 (B-NHL-2009) protocol for risk group 3, modified according to the acute lymphoblastic leukaemia multicentre protocols (MCP-841, S1& S2). Additional file 1: S3, S4 and S5 provide the risk group and drug dosage details. Patient 3 received chemotherapy according to the protocol of the Chinese Children’s Cancer Group study ALL-2015 (CCCG-ALL-2015). Prophylactic intrathecal injections were administered for central nervous system (CNS) involvement (S6); BM samples were obtained and evaluated at different TP as protocols required. FCM monitored BM smears and MRD, and RT-PCR was utilised to verify the results.

### Literature review

Mature BAL patients with KMT2Ar in the literature were retrieved from PubMed, the Web of Science and China national knowledge infrastructure (CNKI). The keyword is “mature lymphocytic leukaemia” or “lymphoma” and “paediatrics”. Data on clinical characteristics, laboratory findings, treatment, and prognosis were collected and analysed.

## Results

### Clinical and lab findings

**Fig. 1:**
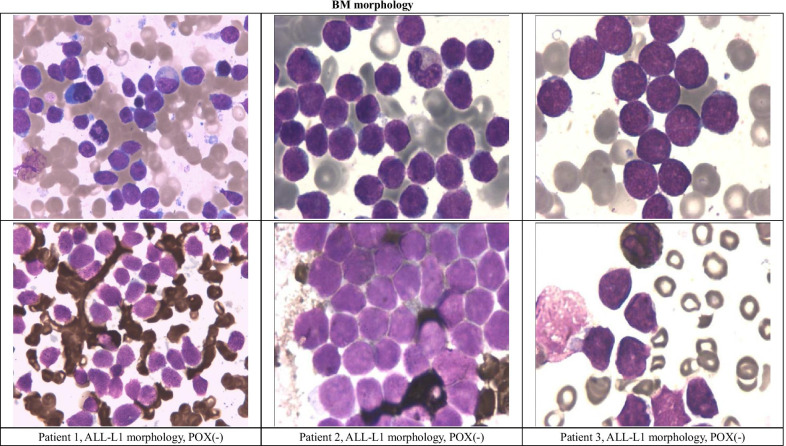
BM morphology

**Fig. 2:**
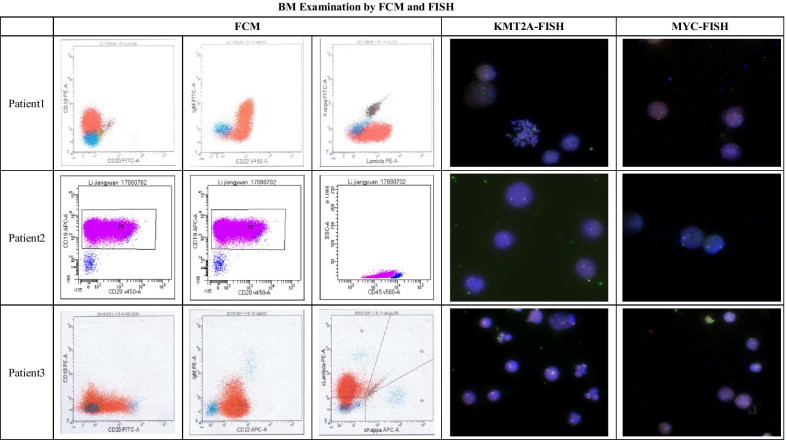
BM examination by FCM and FISH

A total of 198 newly diagnosed B-ALL patients, including 21 BAL patients, were admitted to CHCMU and CWCCH between January 2017 and November 2019, and 3 BAL patients with MLL transcripts were identified, accounting for 1.37% of the B-ALL population. The clinical and laboratory findings for the three reported patients are shown in Table 1. BM samples were obtained at diagnosis, and the results of the BM examinations are listed in Table 2, Figs. 1 and 2.

**Table 1:** Clinical and laboratory findings of reported patients

Pt	Gender/age (m)	Clinical manifestations	WBC (× 10^9^/L)	Hb (g/L)	PLT (× 10^9^/L)	Blast	LDH (^U^/L)
1	Male/8	Paleness and petechiae	373.4	91	38	0.86	367
2	Male/24	Paleness, petechiae, hepatomegaly and abdominal lymphadenopathy	92.05	53	168	0.90	598
3	Male/12	Fever, paleness, petechiae, hepatosplenomegaly, lymphadenopathy, parotid and renal involvement	150.02	66	10	0.76	4059

**Table 2:** FCM, FISH, and PCR results for reported patients

Pt	Morphology	FCM (%)											Chromosomal karyotype	FISH		RT-PCR
		CD10	CD19	CD20	cCD79a	cIgM	cCD22	nuTDT	sIgM	CD22	Kappa	Lambda		KMT2Ar	MYC	
1	ALL-L1	53.1	97.9	0	43.2	28.1	61	0	27.2	83.7	0	65.5	46,XY(20)	53% +	Neg	KMT2A-MLLT3+
2	ALL-L1	88.1	99.8	28.8	97.6	75.1	69.4	0	86.1	99.1	0	84.8	46,XY(20)	90% +	Neg	KMT2A-MLLT3+
3	ALL-L1	9.1	100.0	29.2	89.2	20.4	48.6	0	9.2	904	0	87.7	46,XY,t(?9;11)(p21;q23)(10)/46,XY(10)	94% +	Neg	KMT2A-MLLT3+

### Treatment and prognosis

Patient 1 and Patient 2 were treated with the B-NHL-2009 protocol, and Patient 3 was treated with the CCCG-ALL-2015 protocol. They achieved complete remission (CR) according to morphological, FCM and molecular examination after one chemotherapy course. Allogenic haematopoietic stem cell transplantation (allo-HSCT) was considered by clinicians and was refused by the patients’ parents. A total of six chemotherapy courses were completed for Patient 1 and Patient 2; as of the last follow-up in January 2020, these two patients had a CR status, and their event-free survival (EFS) times were 32 and 29 months, respectively. Chemotherapy was expected to continue for Patient 3, and the EFS time was three months.

### Literature review

The literature, including Case reports and retrospective analysis, was searched in the abovementioned databases, and 12 articles involving 24 patients suffering from BAL with KMT2Ar were found. Clinical and laboratory findings, BM examination results, and the treatment and prognosis of these cases in the literature reports are listed in Tables 3, 4 and 5 [8, 9, 11–21].

**Table 3:** Clinical and laboratory findings of BAL with KMT2Ar according to the literature review

Pt	Reference	Age (m)	Gender	Involved organs	WBC (× 10^9^/L)	Hb (g/L)	PLT (× 10^9^/L)
1	13	96	F	Spleen	43.9	80	6
2	14	23	F	Liver, renal, CNS	87	75	10
3	15	13	M	Lymph node, spleen	60.1	83	63
4	15	12	F	Lymph node, spleen, liver	31.5	132	261
5	17	11	F	–	6.79	57	57
6	16	4	M	Skin, testicular	33.2	ND	136
7	16	8	F	Liver, spleen, adenopathy, skin	160.9	ND	19
8	18	23	F	Liver, CNS, renal	87	75	10
9	18	16	F	Spleen, CNS	93.4	62	133
10	18	5	M	Renal, skin	96.5	96	80
11	18	13	F	Liver, spleen, lymph node	24	49	10
12	18	8	M	spleen	14.58	64	167
13	19	1.5	F	Liver, spleen	329	ND	ND
14	20	4	M	–	117.2	94	54
15	11	108	M	Spleen	1.8	72	45
16	8	3	M	Liver, spleen and renal	857	88	46
17	8	6	M	Liver, spleen and renal, CNS	119	89	85
18	8	6	M	Splenomegaly, skin	11	72	104
19	8	13	F	Lymph node	39	45	11
20	8	18	F	–	68	3	16
21	12	15	F	Liver, renal, lymph node	54	78	41
22	12	4	F	Liver, spleen	295	29	45
23	12	48	F	liver	209	ND	21
24	9	24	M	liver, spleen, lymph node	425.58	56	57

F, female; M, male; ND, no data

**Table 4:** FAB type, FCM and chromosomal karyotyping of BAL with KMT2Ar according to the literature review

Pt	FAB	Gender	CD19	sIgM	Light chain	CD10	CD20	CD22	TdT	CD34	Chromosomal karyotype
1	L1	F	Pos	Pos	L	Neg	Pos	ND	Pos	ND	46,XX(30)/46,XX, 11,t(4,?)(q35;?),t(9,11)(p22;q23),+der(ll)t(l;ll)(q21;q14)(6)
2	L1	F	+	Pos	L	Neg	Pos	Pos	ND	Neg	46,XX(4)/46,XX, t(9;11)(p21-22;q23)(12)
3	L1	M	+	Pos	L	Neg	−/+	Pos	Neg	Neg	Normal
4	L1	F	+	Pos	L	Neg	−/+	Pos	Neg	Neg	Normal
5	Non-L3	F	+	Pos	K	Neg	Pos	Pos	Neg	Neg	46,XX(30)
6	Non-L3	M	+	Pos	L	Neg	−/+	Pos	Neg	Neg	Normal
7	Non-L3	F	+	Pos	L	Neg	−/+	Pos	Neg	Neg	Normal
8	Non-L3	F	+	Pos	L	Neg	−/+	Pos	Neg	Neg	46,XX[4]/46,XX, t(9;11)(p21–22;q23)[12]
9	Non-L3	F	+	Pos	K	Pos	−/+	Pos	Neg	Neg	46,XX[30]
10	Non-L3	M	+	Pos	L	Neg	−/+	Pos	Neg	Neg	46,XX[20].ish add(11)(q23)[20
11	Non-L3	F	+	Neg	L	Neg	ND	Pos	Neg	Neg	46,XX[14].ish t(9;11)(p22;q23)
12	Non-L3	M	+	Pos	K	Neg	Neg	Pos	Neg	Neg	46,XY[10].ish t(9;11)(p22;q23)[15]
13	L1	F	+	Pos	K	Pos	ND	ND	Neg	Neg	46,XX
14	L1	M	+	Pos	Pos	Pos	Neg	Pos	Neg	Neg	46,XY[16]/46,XX,t(9;11;11)(p22;q23;p11.2)[4]
15	ALL-L3	M	−/+	Pos	Pos	Pos	−/+	Pos	−/+	Neg	46,XY,t(11;15)(q23;q15)[10]/46,XY[10]
16	L1	M	+	Pos	L	Neg	Pos	Pos	Neg	Pos	No metaphases
17	Non-L3	M	+	Pos	L	Pos	Pos	ND	Neg	Neg	46,XY,der(2),t(10;11)(p12;q23)[5]
18	L1	M	+	Pos	L	Neg	Neg	Pos	Neg	Neg	46–48,XY,+11,del(11)(q23),
19	L1	F	+	Pos	L	Pos	Neg	Pos	Pos	Pos	46,XX,add(9)(p24),ins(10;11)
20	L1	F	+	Pos	L	Pos	Neg	Pos	Pos	Pos	46,XX[20]
21	L1	F	+	Pos	ND	Pos	Pos	ND	ND	Neg	No metaphases
22	L1	F	+	Pos	ND	Pos	Pos	Pos	Neg	Neg	Normal
23	L1	F	+	Pos	ND	Pos	Neg	Pos	ND	Neg	ND
24	L2	M	+	Pos	L	Neg	Neg	ND	Neg	Neg	46,XY(3)

ND: No data; Pos: Positive; Neg: negative; L: Lambda chain; K: Kappa chain; Normal: reported normal chromosomal karyotype, but exact data were not presented

**Table 5:** FISH, AB type, FCM and chromosomal karyotyping of BAL with KMT2Ar according to the literature review

Pt	KMT2A-FISH	MYC-FISH	Transcript^a^	Protocol	Treatment response	HSCT	Relapse	EFS (m)
1	ND	ND	ND	VLP	Refractory	Refractory	Refractory	9 (OS)
2	Pos	ND	KMT2A +	LMB89	CR	0	BM, CNS	9
3	Neg	Neg	ND	COG-ALL-1961	CR	No	No	9
4	Pos	Neg	ND	CCG-ALL-1991	CR	No	No	4
5	Pos	Neg	ND	Infant ALL	CR	No	No	4
6	Pos	Neg	ND	Interfant-99	CR	No	BM, TL	4
7	Pos	Neg	ND	Interfant-99	CR	4/6 matched CBT	BM	6
8	Pos	Neg	ND	LMB89 trial	CR	No	BM, CNS	6
9	ND	Neg	ND	EORTC 02	CR	No	BM, CNS	6
10	Pos	Neg	KMT2A-MLLT10	Interfant-99	CR	No	No	24
11	Pos	Neg	ND	FRALLE 2000	CR	No	No	35
12	Pos	Neg	ND	Interfant-99	CR	No	TL	14
13	Pos	ND	ND	ALL like	Died	died	died	1.25 (OS)
14	Pos	Neg	KMT2A-MLLT3	ALL	CR	No	No	8
15	Neg	Neg	Neg	ALL	CR	No	No	48
16	Pos	ND	KMT2A/MLLT5	Interfant-99	CR	No	BM, TL	4
17	Pos	ND	KMT2A-MLLT1	BFM-2004	CR	No	BM, TL	19
18	Pos	ND	KMT2A-MLLT1	Interfant-99	CR	No	BM	21
19	Pos	ND	KMT2A-MLLT10	Interfant-99	CR	No	No	32
20	Pos	ND	KMT2A-MLLT5	ALLIC-09	CR	No	No	21
21	Pos	ND	KMT2A-MLLT3	BFM like	CR	Sible-HSCT	No	108
22	Pos	Neg	KMT2A-MLLT3	KMT2A03	CR	mismatched-CBT	No	72
23	ND	ND	KMT2A-MLLT3	BFM-95	CR	MUD-CBT	No	12
24	ND	Neg	KMT2A-MLLT3	B-NHL-2010	CR	No	CNS	13

ND, no data; Pos, positive; Neg: negative; HSCT, haematopoietic stem cell transplantation; CBT, cord blood transplant; MUD, matched unrelated donor; BM, bone marrow; TL, testicular leukaemia; CNS, central nervous system; EFS, event-free survival; OS, overall survival

^a^Transcripts of KMT2Ar were detected by RT-PCR except for patient 2, who was tested by Northern Blot

Data from our hospital and literature about 27 patients suffering from BAL with KMT2Ar were collected and analysed. 13 males and 14 females were enrolled, and the average and the median age at diagnosis were 19.5 ± 4.95 months old and 12 months old, respectively (ranging from 6 weeks to 9 years); 14 (51.85%) and 24 (88.89%) patients were ≤ 1 and ≤ 2 years of age, respectively. Renal, testicular, CNS and skin involvement at diagnosis were present in 6, 1, 4 and 3 patients, respectively. The average white blood cell (WBC) and platelet (PLT) counts and haemoglobin (Hb) levels were 87 ± 35.24 × 10^9^/L, 69.96 ± 5.38 × 10^9^/L and 65.12 ± 12.34 g/L, respectively.

Even though BAL is typically associated with the FAB-L3 morphology, in our research, 26 (96.30%) of the 27 patients showed non-ALL-L3 morphology, and one patient presented with ALL-L3 morphology. FCM confirmed the mature B-ALL phenotype in all 27 patients. Expression of CD19, CD22, and sIgM with light-chain restriction was detected, and TdT and CD34 did not exist in most cases. Expression of CD20 was found in 25 patients. Interestingly, negative or suspicious expression of CD20 was found in 16 (64%) patients, and positive expression of CD20 was detected with a monoclonal antibody in 9 (36%) patients. Although 2 of 3 patients in our report presented with positive CD20 expression, the expression level of CD20 was lower than 30%.

Chromosomal karyotype results were reported for 26 patients, while two patients had no metaphase chromosomes to be analysed; 11q23-related abnormal karyotypes were found in 11 patients. FISH of KMT2Ar was reported in 23 patients, and 22 (95.65%) cases were positive. The results of the detection of KMT2Ar transcripts were presented for 16 patients. Fourteen patients were positive and were identified as having KMT2A-MLLT3 (Formerly MLL-AF9, 6 cases), KMT2A-MLLT5 (Formerly MLL-AF1, 2 cases), KMT2A-MLLT10 (Formerly MLL-AF10, 2 cases), and KMT2A-MLLT1(also called MLL-ENL, 2 cases), while one patient did not have an exact result. FISH may be the most accurate tool for the detection of KMT2Ar. The t (8;14) and its variant were not detected by karyotyping; FISH detected MYCr in 17 patients, and the results were negative.

**Fig. 3:**
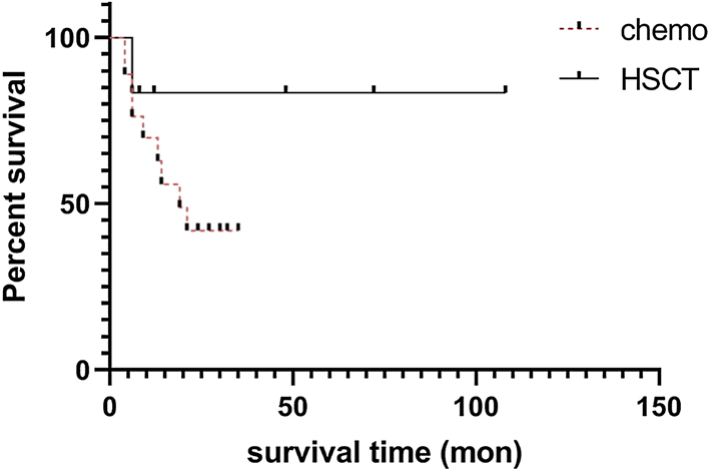
Survival curves of patients. Chemo, chemotherapy; HSCT, haematopoietic stem cell transplantation. Survival analysis by the Kaplan–Meier method and the survival curve of the whole group. Prospective event-free survival (pEFS) in patients who received allogeneic haematopoietic stem cell transplantation (allo-HSCT) was higher than that in patients who received chemotherapy alone (83.33% vs 41.91%)

The 27 patients who received chemotherapy included patients treated with ALL-like (12 cases), BL (8 cases) or Interfant-99 [21] (7 cases) protocols. One patient succumbed to sepsis, one patient presented with refractory status, 25 patients achieved CR, and the CR rate was 92.59%. Besides, six patients received allo-HSCT, 1 (16.67%) patient relapsed six months later, and the prospective 2-yr EFS (pEFS) was 83.33%, as reported in the literature. Nineteen patients subsequently received chemotherapy according to the Interfant-99 (6 cases), BL (6 cases) or ALL-like (7 cases) protocols, and 9 (47.37%) of them relapsed. The 2-yr pEFS was 41.91% (Fig. 3). Four patients in the Interfant-99 and BL groups relapsed, and the 2-yr pEFS in these groups were 40% and 33.33%, respectively. One patient in the ALL-like group relapsed and died after five months of follow-up [22].

## Discussion

BAL has been described as an uncommon subtype of B-ALL; it presents with a unique immunotype characterised by the expression of pan-B-cell markers (such as CD10, CD19, CD20, cCD79a.) and sIgM with light-chain restriction, whereas pB-ALL with surface light-chain immunoglobulin restriction has also been reported [22, 23]. The clinical features, biological characteristics, treatment and prognosis of BAL are similar to those of BL, since in the literature has BL, we compared BAL and BL [2, 7]. BAL patients often show an ALL-L3 FAB morphology; like BL, BAL is characterised by MYC translocations (chromosome 8q24) to an immunoglobulin gene locus, and the MYC gene overexpression was detected in most cases [2, 24]. For patients treated with an intensive short course of chemotherapy, the EFS of BAL and BL has exceeded 90% [24].

KMT2A genes, lysine-specific methyltransferase 2A -related genes, occur in 2.5–5% of paediatric ALL patients and 70% of infant ALL patients [2]. KMT2Ar genes’ presence is often correlated with the phenotype of pB-ALL and leads to a worse prognosis [1, 2], but the presence of KMT2Ar in BAL is a distinctive molecular biological feature, and patients’ prognosis of KMT2Ar is unclear.

We reviewed the literature in databases, and a total of 27 patients, including the three patients described in our manuscript, were found. These three BAL patients with KMT2Ar have unique clinical manifestations and laboratory findings compared with pB-ALL and BL patients. Infant leukaemia patients comprised half of these patients, and most of these patients (24/27) were ≤ two years of age at onset, whereas the median ages of pB-ALL and BL patients were 9 and 2–5 years of age, respectively. Renal, CNS and skin involvement at diagnosis were not unusual and were present in 6, 4, and 3 cases, respectively, in patients with BAL with KMT2Ar; however, renal involvement is not rare in BAL, and CNS or skin involvement is uncommon in both pB-ALL and MAL [1, 6]. Although the reported patients were classified as having a BAL phenotype by FCM, overexpression of CD19 was detected in most cases, and expression of CD20 was not detected; nevertheless, coexpression of CD20 and CD19 is common in BL patients [2]. It has been revealed that rituximab [25], an anti-CD20 monoclonal antibody resulting in the selective depletion of B lymphocytes, was unsuitable for treating these patients; any patient who appeared to be refractory or relapsed may benefit from chimeric antigen receptor T cell (CAR-T) immunotherapy targeting the overexpression of CD19 [26].

BL is often associated with t(8;14)(q24;q32) or its variants, and MYCr is detectable in more than 95% of the BL population [2]. Translocation is the essential driver of the MYC gene’s overexpression, and activation of the MYC gene leads to cell cycle progression, inhibition of differentiation, the promotion of cell proliferation and genomic instability and the activation of endogenous apoptotic programmes [25]. However, it is surprising that the MYC gene and its chromosomal translocation were undetectable in the MAL patients with KMT2Ar.

## Conclusions

Standard treatment of BAL patients with KMT2Ar has not yet been established, and BAL patients seemed to be sensitive to chemotherapy, including chemotherapy administered according to the ALL, BL or Interfant-99 protocols (The details are in the Additional file 1). Most of these patients achieved CR after receiving one chemotherapy course, but the prognosis of patients subjected to different treatments was widely divergent. The pEFS was higher in the allo-HSCT group than in the chemotherapy group. Even though two patients described in our report received chemotherapy with the BL protocol and survived more than two years, the prognosis of patients treated with chemotherapy has remained poor, and allo-HSCT should be recommended for patients with CR1 status.

## Supplementary Information

**Additional file 1:** Describe the details of the treatment, include **S1**: inclusion and exclusion criteria of the B-NHL-2009 protocol; **S2**: the staging system of the B-NHL-2009 protocol; **S3**: the risk groups of the B-NHL-2009 protocol; **S4**: treatment planning of the B-NHL-2009 protocol; **S5**: schedule of the B-NHL-2009 protocol; **S6**: schedule of intrathecal injections for CNS involvement.

## Data Availability

The data, including the clinical and laboratory findings and treatment and prognosis data, are listed in the article and the Additional file 1.

## Abbreviations

AFF1: Proteini AF4/FMR2 family member 1 Gene
ALL: Acute lymphoblastic leukemia
allo-HSCT: Allogeneic haematopoietic stem cell transplantation
BAL: Mature B cell acute lymphoblastic leukaemia
B-ALL: B cell acute lymphoblastic leukaemia
BL: Burkitt lymphoma
BM: Bone marrow
B-NHL-2009: B non-Hodgkin lymphoma 2009 protocol
CAR-T: Chimeric antigen receptor T cell
CCCG-ALL-2015: Chinese Children’s Cancer Group study ALL-2015
CD: Cluster of differentiation
CHCMU: Children’s Hospital of Chongqing Medical University
CNKI: China national knowledge infrastructure
CNS: Central nervous system
CR: Complete remission
CWCCH: Chengdu Women’s & Children’s Central Hospital
FAB: French–American–British
FCM: Flow cytometry
FISH: Fluorescence in situ hybridisation
Hb: Haemoglobin
KMT2Ar: KMT2A rearrangement
MCP-841: Acute lymphoblastic leukaemia multicentre protocol
MRD: Minimal residential disease
multiplex RT-PCR: Multiplex nested reverse transcription-polymerase chain reaction
MYC: Myelocytomatosis oncogene
MYCr: MYC rearrangement
KMT2A: Lysine methyltransferase 2A
pB-ALL: Precursor B-ALL
pEFS: Prospective event-free survival
PLT: Platelet
sIgM: Surface immunoglobulin
TP: Time point
WBC: White blood cell
WHO: World Health Organization
