# Supplementary material for: Mature B cell acute lymphoblastic leukaemia with KMT2A-MLLT3 transcripts in children: three case reports and literature reviews
Source: Orphanet J Rare Dis. 2021 Jul 30;16:331. doi: 10.1186/s13023-021-01972-5 (PMC8325316; doi:10.1186/s13023-021-01972-5)
Supplement: Supplementary file 1 — Additional file 1: Describe the details of the treatment, include S1: inclusion and exclusion criteria of the B-NHL-2009 protocol; S2: the staging system of the B-NHL-2009 protocol; S3: the risk groups of the B-NHL-2009 protocol; S4: treatment planning of the B-NHL-2009 protocol; S5: schedule of the B-NHL-2009 protocol; S6: schedule of intrathecal injections for CNS involvement. [file 13023_2021_1972_MOESM1_ESM.docx]

**Supplementary Tables**

**S1 Inclusion and exclusion criteria of B-NHL-2009 protocol**

| Inclusion criteria | Patients who are diagnosed with: Burkitt Lymphoma; Diffused large B cell lymphoma; Primary large B cell lymphoma of mediastinum; Large B cell lymphoma, ALK positive; B cell non Hodgin lymphoma, none of specific. |
| --- | --- |
| Exclusion criteria | Patients who are diagnosed with primary immunodeficiency disease; secondary lymphoma; past history of organ transplant. |

**S2 staging system of B-NHL-2009 protocol^#^**

| Stage | Definition |
| --- | --- |
| I | Single tumor (extranodal) or single anatomical area (nodal), with exclusion of mediastinum or abdomen. |
| II | Single tumor or extranodal with regional node involvement, areas on same side of diaphragm; Primary GI tract tumor, usually in ileocecal area, completely removed by surgery. |
| III | Pathological lesions are found above and below diaphragm; All primary intrathoracic tumor; All extensive primary intra-abdominal disease which cannot be completely removed by surgery; All paraspinal or epidural tumors, regardless of other tumor sites |
| IV | Any of above with initial involvement of CNS and/or bone marrow involvement. |

#: Similar as Murphy Staging System; GI: gastrointestinal; CNS: central nervous system.

**S3 Risk group of B-NHL-2009 protocol**

| Risk group | Definition |
| --- | --- |
| R1 | Stage I or stage II, tumor was removed by surgery, with normal value of LDH. |
| R2 | Stage I or stage II, tumor cannot be completely removed by surgery, or value of LDH ≤ 2N. |
| R3 | Stage III or stage IV, BAL or value of LDH ≥ 2N. |
| R4 | Patients cannot achieve complete remission after 2 courses of chemotherapy. |

LDH: lactate dehydrogenase; N: upper limit of normal value; BAL: matured B cell acute lymphoblastic leukemia.

**S4 Treatment planning of B-NHL-2009 protocol**

| Risk group | course |  |  |  |  |  |
| --- | --- | --- | --- | --- | --- | --- |
| R1 | A | B | A |  |  |  |
| R2 | A | B | A | B | A |  |
| R3 | P+A | BB | AA | BB | AA | BB |
| R4 | P+A | BB | CC | AA | BB | CC |

Cranial radiotherapy is settled for patients with CNS involvement after chemotherapy; Evaluation is arranged after 2 courses of chemo.

**S5 Schedule of B-NHL-2009 protocol**

| Course | Drugs | Dosage | Date of administration |
| --- | --- | --- | --- |
| Induction | Cyclophosphamide | 300mg/m^2^, iv, 2h | Day 1 |
|  | Vincristine | 1.5mg/m^2^ (max 2mg) | Day 1 |
|  | Prednisone | 45mg/m^2^, po | Day 1-7 |
|  | iT | Based on age | Day 1 |
| A/AA | Cyclophosphamide | 800mg/m^2^, iv, 2h | Day 1 |
|  |  | 200mg/m^2^, iv, 2h | Day 2-4 |
|  | Vincristine | 1.5mg/m^2^ (max 2mg) | Day 1, 8, 15 |
|  | Doxorubicin | 20mg/m^2^, iv, 2h | Day 1,2 |
|  | Cytarabine^#^ | 500mg (or 1500mg)/m^2^, iv, 2h, q12h | Day 1 |
|  | Prednisone | 60mg/m^2^, po | Day 1-7 |
|  | iT | Based on age | Group R1: Day 1  Group R2: Day 1, 8 (1^st^ course)  Group R3: Day 1, 8 |
| B | Ifosfamide | 1200mg/m^2^, iv, 2h | Day 1-5 |
|  | Etoposide | 60mg/m^2^, iv, 2h | Day 1-3 |
|  | Methotrexate | 300mg/m^2^, iv, 3h | Day 1 |
|  | Vincristine | 1.5mg/m^2^ (max 2mg) | Day 8 |
|  | Prednisone | 60mg/m^2^, po | Day 1-7 |
|  | iT | Based on age | Day 1 |
| BB | Ifosfamide | 1200mg/m^2^, iv, 2h | Day 1-5 |
|  | Etoposide | 60mg/m^2^, iv, 2h | Day 1-3 |
|  | Methotrexate | 3000mg/m^2^, iv, 24h | Day 1 |
|  | Tetrahydrofolate^&^ | 15mg/m2, im, q6h | after methotrexate |
|  | Vincristine | 1.5mg/m^2^ (max 2mg) | Day 8 |
|  | Prednisone | 60mg/m^2^, po | Day 1-7 |
|  | iT | Based on age | Day 1, 8 |
| CC | Cisplatin | 100mg/m^2^, iv, 2h | Day 1 |
|  | Dexamethasone | 12.5mg/m^2^, po | Day 1-5 |
|  | Etoposide | 100mg/m^2^, iv, 24h | Day 3-5 |
|  | Doxorubicin | 30mg/m^2^, iv, 2h | Day 1 |
|  | iT | Based on age | Day 1, 8 |

iT: intrathecal injection; #: course of A or AA, dosage of cytarabine is 500mg/m^2^ or 1500mg/m^2^ respectively; &: Administration of tetrahydrofolate begins at 42h after methotrexate, serum level of methotrexate is monitored at 48h, and tetrahydrofolate is withdrawn till serum level of methotrexate ≤1μmol/ml.

**S6 schedule of intrathecal injection**

| Age | Methotrexate | Cytarabine | Dexamethasone |
| --- | --- | --- | --- |
| <12m | 6 mg | 15 mg | 2.5 mg |
| 12-36m | 9 mg | 25 mg | 2.5 mg |
| ≥ 36m | 12 mg | 30 mg | 5mg |
